# p53 Acts as a Co-Repressor to Regulate Keratin 14 Expression during Epidermal Cell Differentiation

**DOI:** 10.1371/journal.pone.0041742

**Published:** 2012-07-24

**Authors:** Bi-He Cai, Pei-Ching Hsu, I-Lun Hsin, Chung-Faye Chao, Mei-Hua Lu, Hwang-Chi Lin, Shih-Hwa Chiou, Pao-Luh Tao, Jang-Yi Chen

**Affiliations:** 1 Department of Biology and Anatomy, National Defense Medical Center, Taipei, Taiwan, Republic of China; 2 Division of Plastic Surgery, Shin Kong Wu Ho-Su Memorial Hospital, Taipei, Taiwan, Republic of China; 3 Department of Medical Research and Education, Taipei Veterans General Hospital, Taiwan, Republic of China; 4 Division of Mental Health and Addiction Medicine, Institute of Population Health Sciences, National Health Research Institutes, Miaoli, Taiwan, Republic of China; 5 Institute of Biomedical Sciences, Academia Sinica, Taipei, Taiwan, Republic of China; 6 Institute of Medical and Molecular Toxicology, Chung Shan Medical University, Taichung, Taiwan, Republic of China; Peking University Health Science Center, China

## Abstract

During epidermal cell differentiation, keratin 14 (K14) expression is down-regulated, p53 expression varies, and the expression of the p53 target genes, *p21* and *14-3-3σ,* increases. These trends suggest that the relative transcriptional activity of p53 is increased during epidermal cell differentiation. To determine the relationship between K14 and p53, we constructed K14 promoters of various sizes and found that wild-type p53 could repress the promoter activity of all of the K14 promoter constructs in H1299 cells. K14-p160 contains an SP1 binding site mutation that prevents p53 from repressing K14 expression. Using a DNA affinity precipitation assay, we confirmed that p53 forms a complex with SP1 at the SP1 binding site between nucleotides -48 and -43 on the K14 promoter. Thus, our data indicate that p53 acts as a co-repressor to down-regulate K14 expression by binding to SP1. Next, we used a 12-O-tetradecanoylphorbol-13-acetate (TPA)-induced epidermal cell differentiation model to examine the inhibition of K14 expression caused by increased p53 activity. Human ovarian teratocarcinoma C9 cells were treated with TPA to induce differentiation. Over-expression of the dominant negative p53 mutant ΔTAp53, which inhibits p53 activity, prevented the TPA-induced K14 down-regulation in C9 cells. Furthermore, treatment of normal primary human foreskin keratinocytes (PHFK) with the p53 inhibitor pifithrin-α (PFT-α) showed that the inhibition of p53 activity relieves K14 repression during epidermal cell differentiation. Finally, we found that TPA induces the phosphorylation of p53 at residue 378, which enhances the affinity of p53 to bind to Sp1 and repress K14 expression.

## Introduction

The transcription factor p53 is a tumor suppressor gene that regulates cell proliferation [1], [2]. In different keratinocyte models, which typically involve calcium (Ca^2+^)-induced epidermal cell differentiation, the expression level of p53 varies depending on the cell line. In cultured human foreskin keratinocytes, the mRNA and protein levels of p53 are down-regulated [3]. However, the p53 mRNA levels in HaCaT keratinocytes do not change [4], whereas the p53 protein expression increases in human neonatal foreskin keratinocytes [5]. Despite the inconsistencies in p53 expression levels, p53 downstream genes such as p21 [3], [5] and *14-3-3σ* [3] are activated after epidermal cell differentiation, suggesting that relative p53 activity is increased during epidermal cell differentiation. p63, another member of the p53 family that is highly expressed in the basal layer of the epidermis [6], [7]. ΔNp63α is the highest expressing p63 isoform and acts as a stem cell marker in the epidermis [8]. Furthermore, all p53 family members are capable of binding to each other through their native oligomerization domain [9]. Because ΔNp63α has a dominant negative effect on p53 [10], ΔNp63α expression inhibits p53 function in the basal cells of the epidermis [3]. After epidermal cell differentiation, the level of ΔNp63α decreases dramatically, and p53 inhibition is relieved from ΔNp63α resulting in the activation of p53 target genes [3]. Keratin 14 (K14) is an epidermal basal cell marker whose expression is decreased during epidermal cell differentiation [11]. The transactivation isoform (TA) and the ΔN isoform of p63 can directly and indirectly enhance K14 expression [12], [13], [14], [15]. Therefore, ΔNp63α depletion is a prerequisite for K14 repression during epidermal cell differentiation. Based on this information, we hypothesized that activated p53 may also be involved in the regulation of K14 gene expression during epidermal cell differentiation. We used the human ovarian teratocarcinoma (HOTC) C9 cell line as a model to study keratinocyte differentiation. C9 cells showed the ability to uptake melanosomes when co-cultured with melanocytes [16]. C9 cells have also been used to grow keratinized tumors in nude mice [17]. 12-O-tetradecanoylphorbol-13-acetate (TPA) is a molecule with a structure similar to diacylglycerol (DAG) and is able to induce Ca^+2^ release from the endoplasmic reticulum (ER), resulting in the activation of protein kinase C (PKC) and the differentiation of keratinocytes [18]. Thus, we treated C9 cells and primary human foreskin keratinocytes (PHFK) with TPA to determine how p53 down-regulates endogenous K14 expression.

## Results

### p53 Represses the Proximal Promoter Region of K14

**Figure 1:**
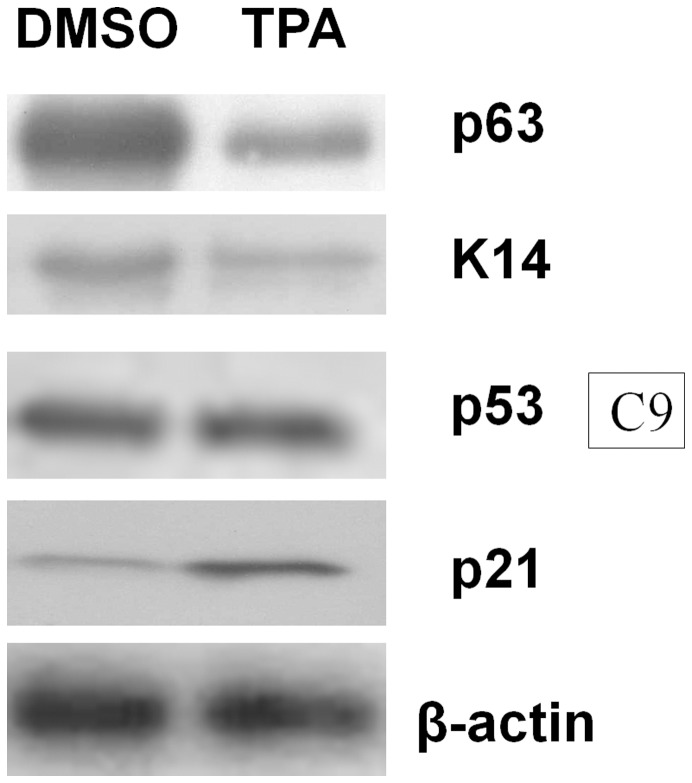
p53 activity is up-regulated after TPA treatment in C9 cells. C9 cells were treated with DMSO (control) or TPA (100 ng/mL). Compared to the DMSO-treated samples, TPA treatment reduced p63 and K14 expression. Although p53 levels did not vary based on TPA treatment, the p53 target gene p21 was induced following TPA treatment. β-actin expression was used as a loading control. Each well was loaded with 20 µg of total protein extract.

**Figure 2:**
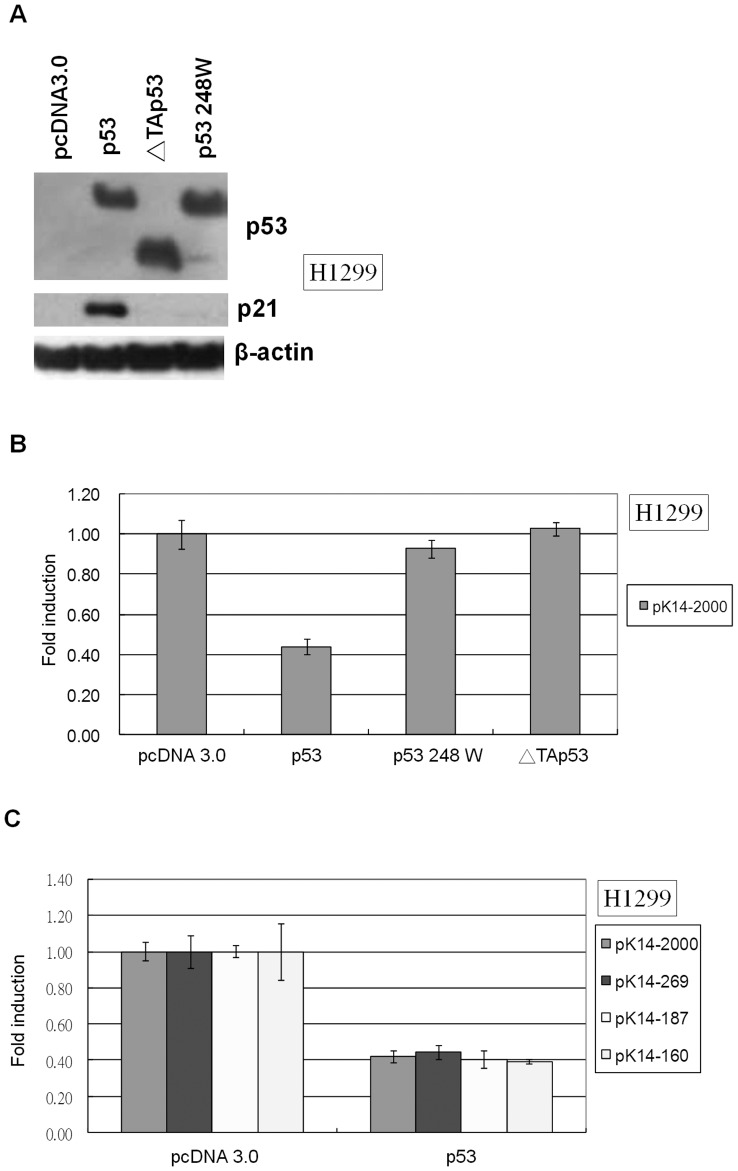
An active form p53 can repress K14 promoter within the first 160 base pairs of the K14 promoter. (A) H1299 cells were transfected with pcDNA 3.0, wild-type p53, mutant p53 248W, or ΔTAp53 expression vectors. Wild-type p53 up-regulated p21 expression, but p53 248 W and ΔTAp53 did not. β-actin expression was used as a loading control. Each well was loaded with 20 µg of total protein. (B) H1299 cells were co-transfected with the pK14-2000 promoter vector and pcDNA 3.0, wild-type p53, mutant p53 248 W, or ΔTAp53 expression vectors. The levels of luciferase promoter activity were normalized to that of pcDNA 3.0, which was set to 1. Wild-type p53 down-regulated pK14-2000 promoter activity, but p53 248 W and TAp53 did not. (C) H1299 cells were co-transfected with pK14-2000, pK14-269, pK14-187, or pK14-160 promoter vectors and pcDNA 3.0 or wild-type p53 expression vectors. The levels of luciferase promoter activity were normalized to that of pcDNA 3.0, which was set to 1. The activity of all K14 promoter truncations was down-regulated by p53.

After treating C9 cells with TPA, we observed a decrease in ΔNp63α and K14 levels (Fig. 1). p53 protein levels remained unchanged following TPA treatment; however, the expression of the p53 target gene p21 was induced by TPA (Fig. 1). These results suggest that TPA treatment induces p53 activity in C9 cells. To determine the impact of p53 activation on K14 expression, p53 null H1299 cells were transfected with wild-type p53, the DNA binding domain mutant p53 248 W, or the p53 transactivation domain deletion mutant ΔTAp53 (Fig. 2A). We found that only wild-type p53 was able to repress K14 promoter activity (Fig. 2B). In K14 promoter mutants containing deletions of the K14 promoter region, wild-type p53 could inhibit pK14-2000, pK14-269, pK14-187, and pK14-160, suggesting that only activated p53 can repress K14 expression and that this occurs at the proximal promoter region (Fig. 2C).

### p53 Acts as a Co-repressor with SP1 to Repress K14 Expression

**Figure 3:**
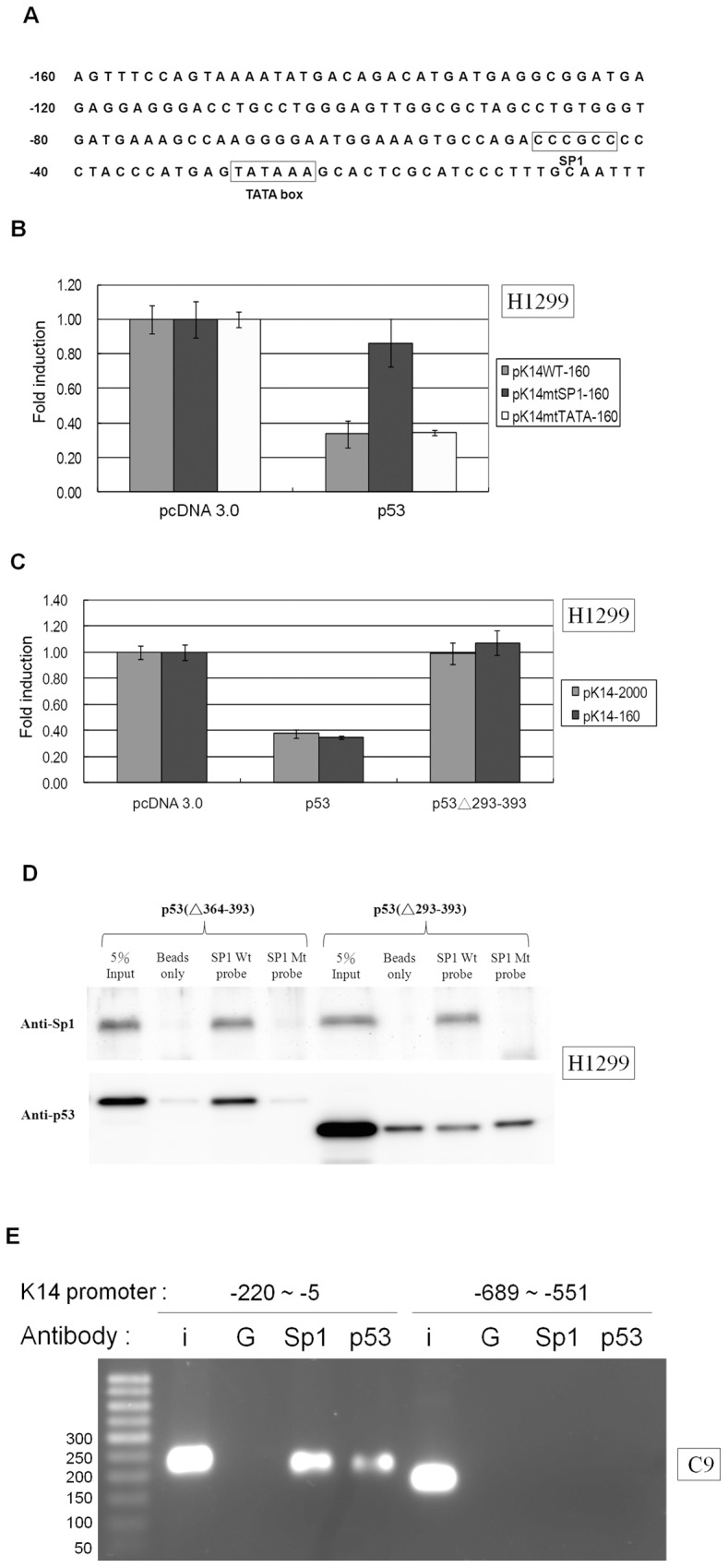
p53 represses the K14 promoter indirectly by associating with transcription factor SP1. (A) The sequence map of the first 160 base pairs of the K14 promoter. No p53 consensus binding site was found within the K14 promoter based on a search for the following typical p53 consensus binding site: PuPuPuC(T/A)(T/A)GPyPyPy-N(013)-PuPuPuC(T/A)(T/A)GpyPyPy; N(0–13) indicates a spacer of 0 to 13 bases. There is an SP1 binding site between base pairs -48 and -43 and a TATA box binding site located between base pairs -29 and -24. The K14 promoter sequence from the GenBank database [GenBank: HSU11076] is shown, and the transcription initiation site refers to the NCBI reference sequence of the human K14 gene [GenBank: NM_000526.4]. (B) H1299 cells were co-transfected with wild-type K14 (pK14WT-160), the TATA site mutant (pK14mtTATA-160), or the SP1 site mutant (pK14mtSP1-160) promoter vectors and pcDNA 3.0 or wild-type p53 expression vectors. The levels of luciferase signal were normalized to the luciferase level of pcDNA 3.0, which was set to 1. p53 was able to repress luciferase expression driven by the pK14WT-160 and pK14mtTATA-160 promoter constructs, but not the pK14mtSP1-160 promoter construct. (C) H1299 cells were co-transfected with pK14-2000 or pK14-160 promoter constructs and pcDNA 3.0, wild-type p53, or p53Δ293-393 expression vectors. The level of luciferase signal from each sample was normalized to the pcDNA 3.0 sample, which was set to 1. p53 but not p53Δ293-393 suppressed luciferase expression driven by the pK14-2000 and pK14-160 promoters. (D) DAPA was used to purify the nuclear protein extract from H1299 cells transfected with the p53Δ363-393 or p53Δ293-393 expression vector. Nuclear proteins were incubated with oligonucleotide probes corresponding to base pairs -53 to -38 of the K14 promoter with a wild-type SP1 binding site CCCGCC or with an SP1 binding site mutant GGTACC. Nuclear proteins that co-precipitated with the probes were blotted with anti-SP1 or anti-p53 antibodies. The SP1 binding site on the K14 promoter co-precipitated with SP1 and p53Δ363-393 but not p53Δ293-393. The SP1 binding site mutant probe was unable to pull down SP1, p53Δ363-393, or p53Δ293-393. “5% input” denotes the positive loading control for nuclear protein, and “beads only” denotes the non-probed negative control. (E) In the ChIP assay, Sp1 and p53 antibodies immunoprecipitated base pair -220 to -5 of the K14 promoter in C9 cells. Non-specific IgG antibody was included as the negative control. IgG, Sp1 or p53 antibody was unable to immunoprecipitate the K14 promoter fragment from base pair -689 to -551. (I: input; G: IgG).

Sequence analysis revealed no canonical p53 binding site within the 160-bp region of the K14 promoter directly upstream from the start codon (Fig. 3A). Therefore, we considered the possibility that p53 might act as a co-repressor by associating with other transcription factors to repress K14 expression. p53 has been shown to repress certain genes in an SP1 binding site-dependent [19], [20] and TATA box-dependent manner [21], [22]. Furthermore, an SP1 binding site and a TATA box were found within pK14-160 (Fig. 3A). Therefore, we created a TATA box mutant and an SP1 binding site mutant within the first 160 nucleotides of the K14 promoter region to determine the effect of p53 regulation. The SP1 mutant but not the TATA mutant was able to reverse the repressive effect of p53 on K14 expression (Fig. 3B). p53 is able to interact with SP1 through its C-terminal domain within amino acids 293-393 [23]. Mutant p53Δ293-393 was unable to inhibit K14 expression suggesting that SP1 binding by p53 is necessary (Fig. 3C). An SP1 site on the K14 promoter was used as a probe to pull down p53 through a DNA affinity precipitation assay (DAPA). The p53 C-terminal 363-393 region contains several basic amino acids, which may accommodate non-specific binding to negatively charged DNA molecules [24]. Therefore, we used the basic domain deletion p53 mutant p53Δ363-393 as a specific binding molecule for p53 in the DAPA assay [25]. The SP1-specific probe on the K14 promoter was able to pull down both SP1 and p53Δ363-393 but not p53Δ293-393. Furthermore, the SP1 mutant probe was unable to pull down SP1, p53Δ363-393, and p53Δ293-393 (Fig. 3D). In addition, a ChIP assay revealed that Sp1 and p53 could occupy the proximal but not the distal region of the K14 promoter (Fig. 3E). These results suggest that p53 can associate with SP1 to bind to the SP1 binding site on the K14 promoter.

### A dominant Negative form of p53 can Reverse the p53-mediated Repression of K14 Expression

**Figure 4:**
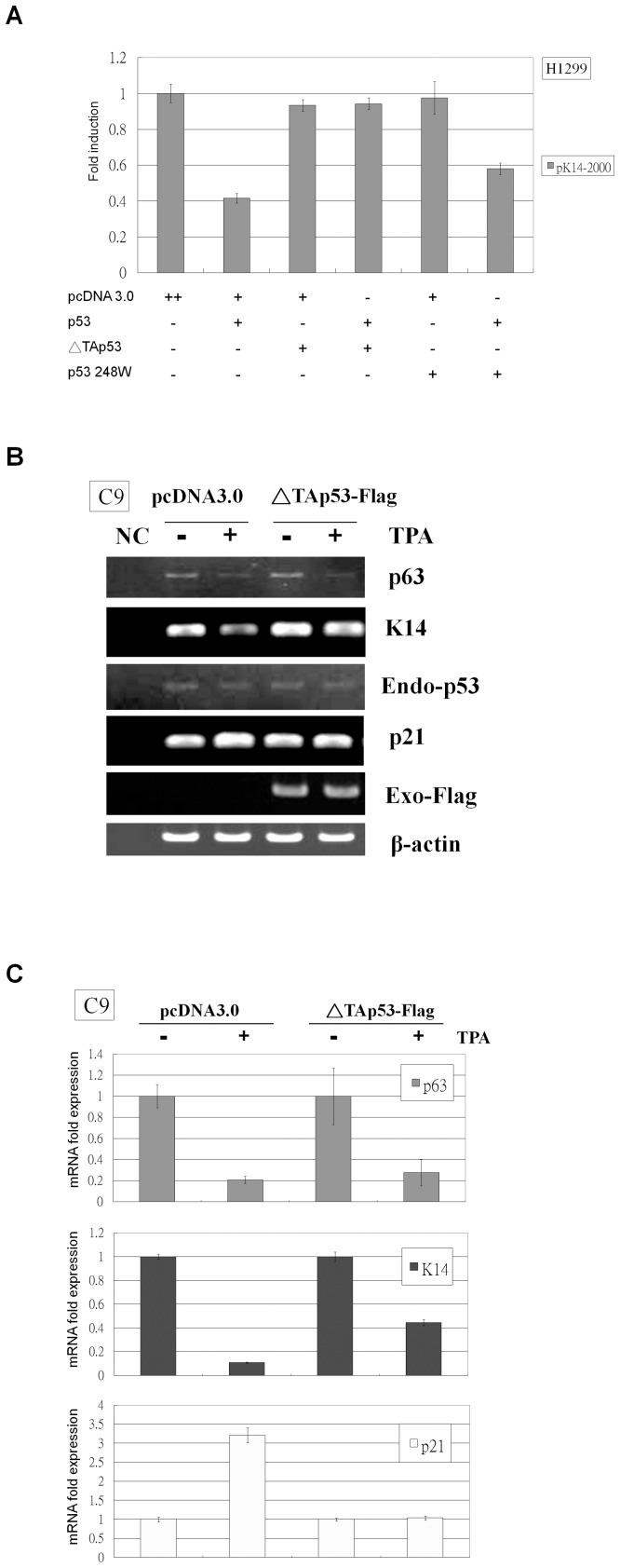
ΔTAp53 relieves TPA-induced K14 suppression in C9 cells. (A) H1299 cells were co-transfected in a three-vector reaction with the pK14-2000 promoter vector, pcDNA 3.0 or wild-type p53 expression vector, and pcDNA 3.0, p53 248 W, or ΔTAp53 expression vector. The levels of luciferase promoter activity were normalized to the pcDNA 3.0, which was set to 1. Wild-type p53 repressed pK14-2000 promoter activity, but this effect was completely reversed when ΔTAp53 was co-transfected or partially reversed when p53 248 W was co-transfected. (B) C9 cells were transfected with pcDNA 3.0 or ΔTAp53-Flag and then were treated with DMSO or TPA. Following treatment, total RNA was isolated from each sample for RT-PCR analysis. p63 mRNA was down-regulated by TPA treatment, but endogenous p53 Mrna levels did not change after TPA treatment. K14 and p21 mRNA expression were down- and up-regulated by TPA, respectively, but these effects were diminished in ΔTAp53-Flag transfected cells.Anti-Flag antibody was used to assess exogenous ΔTAp53-Flag expression, and anti-β-actin antibody was used as a loading control. “NC” denotes the non-cDNA PCR control. (C) Total RNA was prepared from the same treatment conditions as in Figure 4B and was subjected to real-time RT-PCR analysis. p63 was down-regulated by TPA treatment in pcDNA 3.0- and ΔTAp53-Flag-transfected cells. K14 was down-regulated by TPA treatment, but this effect was partially reversed in ΔTAp53-Flag-transfected cells. p21 was up-regulated by TPA treatment, but this effect was fully reversed in ΔTAp53-Flag-transfected cells.

Introducing a dominant negative regulator can effectively inactivate transcription factor activity [26]. As a functional regulator, p53 exists as a tetramer, and mutant p53 tends to be dominant negative because they are capable of associating with wild-type p53 and inactivating p53 function [27]. p53 DNA binding domain mutants such as p53 248 W need to bind to three of the four molecules within the tetramer to inhibit p53 function, but ΔTAp53 is able to inhibit p53 activity by binding to only one of the four molecules within the tetramer [28]. Wild-type p53 and either p53 248 W or ΔTAp53 were co-transfected into H1299 cells to determine which p53 mutant had a larger dominant negative effect on K14 expression. Our results show that p53 248 W is able to partially reverse the down-regulation of K14 by p53, whereas ΔTAp53 completely prevented K14 down-regulation (Fig. 4A). Therefore, ΔTAp53 was used to block p53 activity induced by TPA in C9 cells (Fig. 4B and 4C). TPA down-regulated K14 and p63 mRNA expression and induced p21 mRNA expression (Fig. 4B and 4C). When C9 cells were transfected with ΔTAp53 and then were treated with TPA, p63 expression decreased, but the TPA-induced repression of K14 and the enhancement of p21 were reduced. (Fig. 4B and 4C). These results suggest that the active form of p53 induced by TPA represses K14 expression and increases p21 expression, and these effects can be reversed by the presence of a dominant negative p53 mutant.

### The p53 Inhibitor Pifithrin-α can Relieve the TPA-induced K14 Repression in Primary Human Foreskin Cells

**Figure 5:**
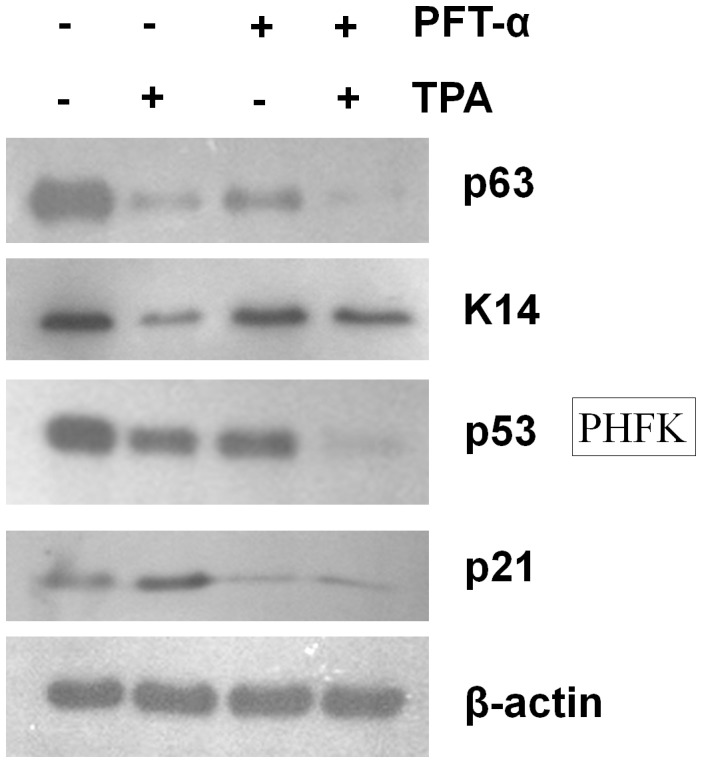
PFT-α reversed TPA-induced K14 down-regulation in PHFK cells. PHFK cells were treated with PFT-α or DMSO and then were treated with TPA or DMSO. Total protein was assayed by western blot. p53 and p63 protein expression was down-regulated upon treatment with PFT-α, and TPA treatment down-regulates the expression of p53 and p63 in non-PFT-α- or PFT-α-treated cells. K14 and p21 protein expression were down- and up-regulated by TPA, respectively, but these effects were relieved in PFT-α pre-treatment cells. β-actin was used as the loading control. Each well was loaded with 20 µg of total protein.

Owing to plasmid transfection to PHFK cells has higher toxicity; we used a chemical inhibitor of p53, PFT-α [29] to determine the effect of p53 on TPA-induced K14 repression in PHFK cells. PFT-α treatment resulted in a decrease in p53 and p63 protein expression in PHFK cells, and TPA was able to down-regulate p53 and p63 expression in non-PFT-α- or PFT-α-treated cells (Fig. 5). K14 protein expression was decreased in cells treated with TPA, but these effects were reversed when the cells were pretreated with PFT-α. Furthermore, p21 protein expression was up-regulated in cells treated with TPA, and this effect was reversed when the cells were pretreated with PFT-α.

### TPA Induces the phosphorylation of p53 residue 378 to repress K14 expression

**Figure 6:**
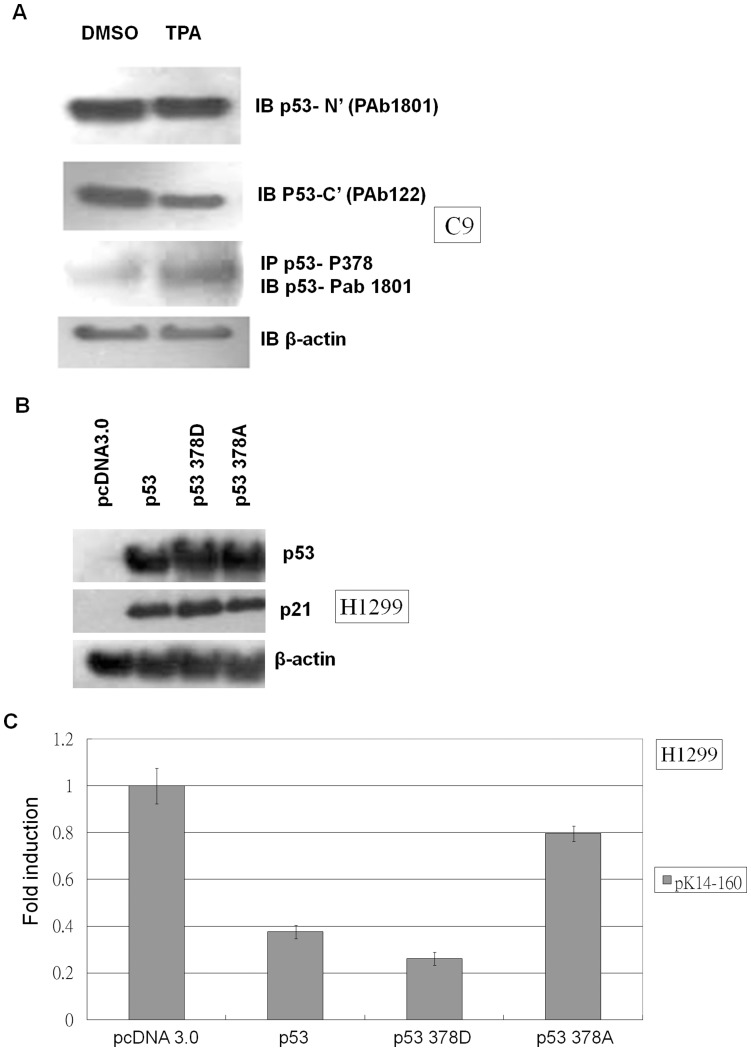
TPA-induced modification of p53 residue 378 moderates the suppression of K14 expression. (A) C9 cells were treated with DMSO or TPA, and total protein was isolated and blotted with different p53 antibodies. p53 levels probed with the N-terminal antibody Pab 1801 were similar for DMSO- and TPA-treated samples. Compared to the DMSO control, p53 levels probed with the p53 C-terminal antibody Pab 122 decreased relative to the TPA-treated samples. However, p53 signal increased in the TPA-treated sample when the sample was first immunoprecipitated with an antibody specific for p53 phosphorylated at residue 378 and then was blotted with p53 Pab 1801. β-actin was used as a loading control. Each well was loaded with 20 µg of total protein. (B) H1299 cells were transfected with pcDNA 3.0, p53, p53 378A, or p53 378D expression vectors and then were analyzed by western blot. Wild-type p53 increased the expression of p21, and the constitutively phosphorylated mimic p53 378D increased p21 expression to a greater extent than the unphosphorylated p53 378A mutant. β-actin expression was used as a loading control. Each well was loaded with 20 µg of total protein. (C) H1299 cells were co-transfected with the pK14-160 promoter vector and pcDNA 3.0, p53, p53 378A, or p53 378D expression vector and then were subjected to a luciferase assay. The fold-change in luciferase promoter activity of each sample was normalized to the pcDNA 3.0 sample, which was set to 1. The constitutively phosphorylated mimic p53 378D mutant repressed K14 promoter activity more than wild-type p53, whereas the unphosphorylated p53 378A repressed the K14 promoter less than wild-type p53.

The p53-specific antibody Pab122 recognizes residues 370-378 unless they become phosphorylated by an event such as epidermal differentiation [30]. Wild-type p53 but not the p53 378A mutant is able to repress the cyclin B1 promoter [31]. Therefore, we harvested protein from TPA-treated C9 cells and subjected the lysates to western blot analysis by probing them with p53 Pab122 and p53 378 phosphorylation-specific antibodies. TPA treatment did not affect p53 levels when lysates were probed with the p53 N-terminal-specific antibody Pab1081 (Fig. 6A). However, TPA treatment reduced the signal obtained from probing C9 cell lysates with p53 Pab122 antibody and increased the signal obtained from probing C9 cell lysates with p53 378 phosphorylation-specific antibody (Fig. 6A). These results suggest that TPA treatment can induce p53 phosphorylation at residue 378. Then, we analyzed the effect of various p53 378 mutants on the regulation of p21 and K14 expression. The constitutively phosphorylated mimic p53 378D was able to increase p21 expression more than the unphosphorylatable p53 378A mutant (Fig. 6B). This indicates that p53 a.a. 378 hyper-phosphorylation leads to a higher level of activity than the hypo-phosphorylated form, and suggests that the phosphorylation of p53 residue 378 might increase p53-mediated suppression of K14. The p53 378D mutant was able to repress K14 promoter activity to a greater extent than wild-type p53, whereas the p53 378A mutant repressed the K14 promoter significantly less than wild-type p53 (Fig. 6C). These results suggest that TPA is able to induce the phosphorylation of p53 residue 378 to promote K14 repression.

### TPA Treatment and Phosphorylation Status of p53 Residue 378 Promotes p53-SP1 Binding

**Figure 7:**
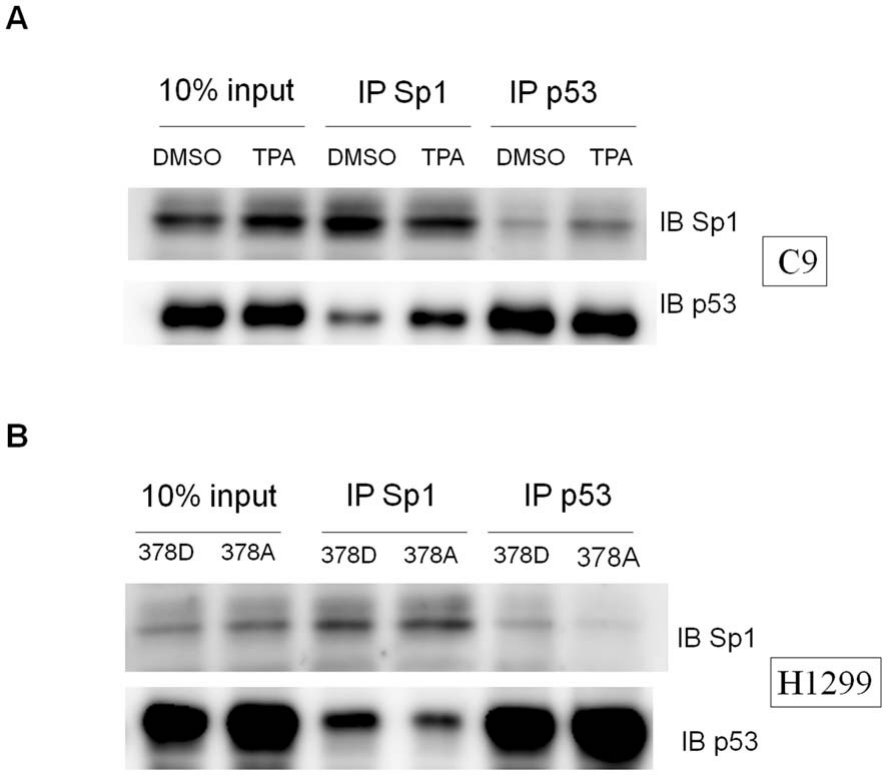
Sp1 and p53 interaction under TPA treatment depends on the phosphorylation status of p53 residue 378. (A) When C9 cells were subjected to TPA treatment, Sp1 could immunoprecipitate p53 significantly more than the DMSO mock-treated sample. p53 could reciprocally immunoprecipitate Sp1 relative significantly in TPA-treated cells more than the DMSO mock-treated sample. The input samples consisted of 10% of the lysate subjected to IP (B) When H1299 cells were transfected of different p53 mutants, the p53 378D mutant was able to immunoprecipitate Sp1 to a greater extent than the p53 378A mutant. Sp1 could reciprocally immunoprecipitate the p53 378D mutant significantly more than the p53 378A mutant.

Our results show that p53 can interact with Sp1 and suppress K14 expression through the Sp1 binding site on the K14 promoter. The deletion of the SP1 interaction domain in p53 prevented the repression of K14 expression. Therefore, the p53 and SP1 protein-protein interaction could be critical to the p53-dependent repression of K14 expression. TPA-induced phosphorylation of p53 at residue 378 might promote p53 binding to SP1 and enhance the repression of K14 expression. Endogenous co-immunoprecipitation of C9 cell lysates revealed increased SP1-p53 interaction upon TPA treatment when compared with mock-treated cells (Fig. 7A). Furthermore, constitutively phosphorylated p53 mimic p53 378D was able to bind SP1 significantly more than the unphosphorylatable p53 378A mutant when overexpressed in H1299 cells (Fig. 7B). These results suggest that TPA promotes the phosphorylation of p53 at residue 378, which could promote the p53 and Sp1 protein-protein interaction.

### p53 Levels Affect Endogenous K14 Expression

**Figure 8:**
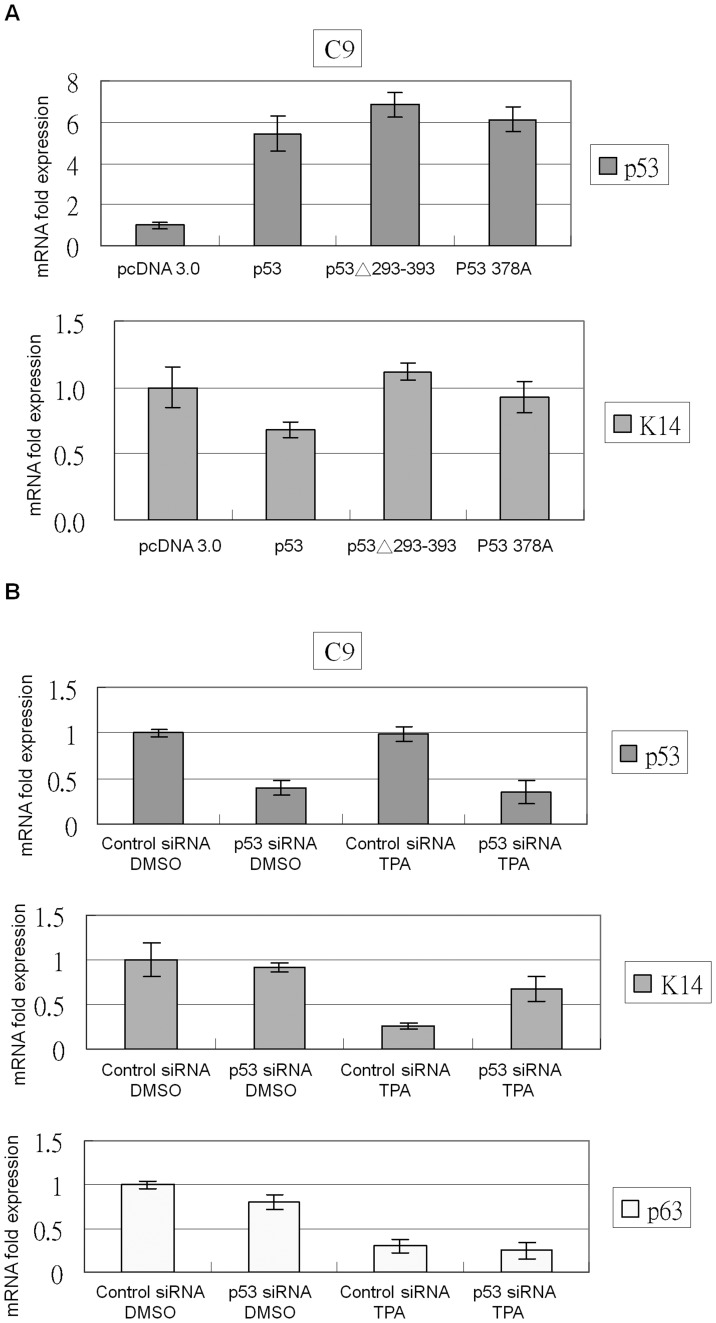
p53 represses K14 expression in the presence of TPA. (A) C9 cells were transfected with various p53 constructs, and total RNA was collected to perform real-time PCR. Wild-type p53 but not pcDNA 3.0, p53 378A, or p53Δ293-393 could inhibit K14 expression. (B) Control or p53 siRNA was transfected into C9 cells, which were then treated with DMSO or TPA. TPA-treated control siRNA-transfected samples showed repression of K14 expression, but K14 repression was reversed in p53 siRNA-transfected cells. Both TPA and p53 siRNA reduced p63 expression.

To evaluate the effect of altering p53 levels on K14 expression, C9 cells were transfected with several p53 over-expression vectors or p53 siRNA constructs. Wild-type p53 but not p53 378A or p53Δ293-393 inhibited K14 expression (Fig. 8A). p53 siRNA transfection in C9 cells did not obviously affect K14 expression, but p63 expression slightly decreased (Fig. 8B). p53 siRNA-transfected cells treated with TPA showed less K14 repression when compared with TPA-treated control siRNA-transfected cells. These results suggest that knocking down p53 reverses TPA-mediated K14 repression.

## Discussion

Our results indicate that only functional wild-type p53 is able to repress K14 expression (Fig. 2A). Furthermore, this regulation is indirect; p53 binds to the SP1 binding site as a co-repressor (Fig. 3B and 3C). ΔTAp53, which is capable of interacting with SP1 [23], was unable to repress K14 expression (Fig. 3B), suggesting that p53 activity is critical for SP1-dependent inhibition of K14 expression. The DNA binding domain mutation p53 248W, which does not affect SP1 binding, not only lost its activity but was also unable to repress K14 promoter function (Fig. 2B). Similarly, p73 isotypes TAp73α and TAp73β, which contain functional TA domains, can repress human telomerase reverse transcriptase (hTERT) expression through indirect binding to SP1 sites on the hTERT promoter. However, the R293H DNA binding domain mutants ΔNp73α, ΔNp73β, and TAp73β are unable to regulate the hTERT promoter [32].

**Figure 9:**
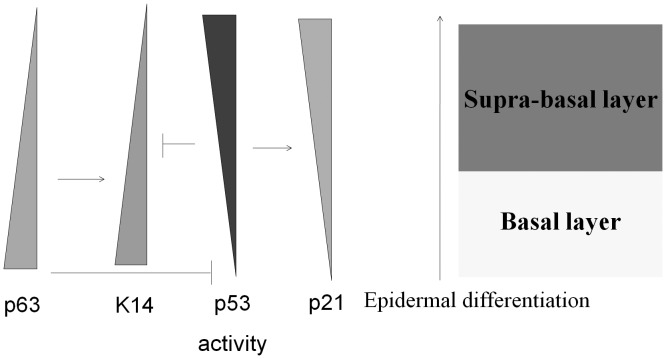
p53 and p63 are involved in the regulation of epidermal differentiation. p63 is used as a stem cell maker because it is persistently expressed in the basal layer of the epidermis and is inhibited during epidermal differentiation. Because p63 only expresses a dominant negative isoform, ΔNp63α, within the epidermal cell basal layer, p53 activity is up-regulated after p63 withdrawn as a result of epidermal differentiation, and p53 downstream targets such as p21 become induced. K14 is a basal layer-expressed keratin subtype and can be up-regulated by p63. During epidermal differentiation, K14 expression is down-regulated by the loss of p63 and is repressed by the activity of p53.

p63 is a key regulator of epidermal development and differentiation [6], [7], [33], [34], but the role of p53 in epidermal differentiation remains unclear. One study suggests that functional p53 over-expression in HaCat cells promotes differentiation faster than control cells [30]. Another study suggests that the HPV E6 oncoprotein is able to degrade p53 and inhibit the differentiation of human keratinocytes [35]. The other study shows that ultraviolet (UV) light induces mouse epidermis differentiation, but this effect is lost in p53 null epidermal tissue [36]. Our results suggest that p53 activation promotes the expression of p21 and the repression of K14 during epidermal differentiation. In addition, the down-regulation of p63 led to a loss of activation function on K14 (Fig. 9). These results suggest a mechanism whereby the replacement of the basal layer expressing K14 with the supra-basal layer containing keratin during the epidermal differentiation is cooperatively mediated by both p53 and p63. Other examples of p53 and p63 cooperation in the epidermis include Notch1 and Hsp70. Notch 1 is primarily distributed in the supra-basal layer of the epidermis [37]. ΔNp63α suppresses Notch1 activity in keratinocytes within the basal layer [38], and p53 can enhance Notch 1 expression after keratinocyte differentiation [36]. Hsp70 is most highly concentrated in the basal layer of the epidermis. [39]. ΔNp63α enhances Hsp70 expression [40], and p53 represses Hsp70 expression. [41]. Based on our data and previous studies, we predict that many keratinocyte-specific markers that are regulated by p53 and p63 during epidermal differentiation will be identified.

p53 represses gene function through both direct and indirect promoter binding. p53 has been shown to act as a repressor by directly binding to a canonical p53 response element [42], [43]. In addition, p53 may act as a co-repressor that indirectly binds to other transcription factor binding sites (i.e., GC box, CCAAT box, and TATA box) to suppress gene expression [44], [45], [46]. There are two mechanisms through which p53 interacts with other transcription factor binding sites and represses genes. First, the binding of p53 to another transcription factor causes the transcription factor to release from its specific binding site [47], [48]. Second, p53 can bind to another transcription factor and recruit histone-modifying enzymes to prevent the recruitment of RNA pol II to the proximal promoter regions [49], [50], [51]. Our results show that p53 interacts with SP1 to associate with a GC box within the K14 promoter (Fig. 3D). The identification of histone modifiers that are recruited by p53 in the K14 proximal promoter region is the next step to give more insight into in the role of p53 involved in K14 repression.

Post-translational modification of p53 is highly dependent upon the desired function of p53 activation. Our study shows that TPA induces the phosphorylation of residue 378 of p53 to promote K14 repression (Fig. 6A and 6C). Furthermore, constitutively activated mutant p53 378D promoted higher p21 expression than the unphosphorylatable p53 378A mutant (Fig. 6B). These results suggest that TPA promotes p53 activity by phosphorylating serine 378, which increases the repression of K14 (Fig. 6B and 6C). Several reports have suggested that the phosphorylation of p53 at serine 378 promotes specific DNA binding affinity [52], [53], [54]. The 14-3-3 family isoforms ε and γ have been shown to enhance the formation of functionally active p53 tetramers [54]. Furthermore, the phosphorylation of p53 residue 378 creates a docking site for the binding of 14-3-3 family members [52], [53]. Moreover, in psoriasis patients, keratinocytes within the epidermis undergo hyper-proliferation, and K14 is heavily expressed in the supra-basal layer [55]. Higher levels of p53 are found in psoriatic plaques when compared with normal skin [56], [57], but phosphorylated and acetylated p53 was not detected [57]. Thus, p53 is highly expressed but may not exist as an active transcription factor in psoriatic plaques. Our results show that only activated p53 is capable of repressing K14 expression (Fig. 2B), which could offer a potential link between inactivated p53 and the abnormal expression of K14 in psoriatic plaques.

Because the ΔNp63α promoter has a p53 response element, using p53 shRNA to knock down p53 expression causes p21 down-regulation as well as ΔNp63α down-regulation [58]. p63 could also be slightly repressed in p53 siRNA transfected C9 cells (Figure 8B). In our study, we blocked p53 activity by using the dominant negative form ΔNp53, which could only block the TPA-induced p21 up-regulation without affecting ΔNp63α expression in mock-treated or TPA-treated C9 cells (Fig. 4B and 4C). Treatment of PHFK cells with the chemical p53 inhibitor PFT-α also caused the down-regulation of p21, p53, and ΔNp63α subjected to mock or TPA treatment (Fig. 5). Although PFT-α has the p53 shRNA-like effect to influence the ΔNp63α expression, PFT-α was also capable of reversing TPA-induced K14 down-regulation (Fig. 5). Therefore, our results strongly suggest that activated p53 repress K14 expression in PHFK cells.

Because p53 expression decreased in PHFK cells but not in C9 cells after TPA treatment (Figs. 1 and 5), we focused primarily on the C9 cell line to study how p53 regulates K14 expression. However, C9 cells are not keratinocytes and thus cannot comprehensively model the role of p53 in the epidermal differentiation pathway. Therefore, we included PHFK cells in our experiments to more closely mimic physiological conditions. After treating PHFK cells with TPA or PFT-α (Figure 5), p53 protein levels decreased. The level of p63 was almost completely lost following TPA treatment, but K14 expression could still be restored by PFT-α within TPA treatment. So we believed that p53 also had the function in repression of K14 expression in PHFK cells. The molecular mechanism of action for PFT-α is not clear, but PFT-α has been reported to inhibit p53 activity by blocking its nuclear transport [59]. PFT-α can reduce doxorubicin-induced p53 protein expression in rat myoblastic cells and etoposide-induced TAp73 and ΔNp73 protein expression in rat testes [60], [61]. In our study, we also found that p53 and ΔNp63α levels decreased upon PFT-α treatment. Sun *et al*. have reported that PFT-α does not affect exogenous poly (I-C)-induced TAp63α-mediated cell death in HUVEC cells [62]. Therefore, PFT-α might have a different effect on various members of the p53 family in different types of cells.

One report mentions that TPA can enhance Sp1-p53 binding in the Jurkat T-cell line [63], and we reproduced this phenomenon in C9 cells (Fig. 7A). p53 can bind to SP1 through the 101 residues at the C-terminus, but p53 mutants harboring a deletion of the last 30 residues can also interact with SP1. Koutsodontis *et al.* [22] have reported that wild type p53 binds SP1 more strongly than p53 in which the last 30 residues have been deleted. Based on our data, one of the residues within this stretch of amino acids that may be responsible for interaction with Sp1 is serine 378, which appears to require phosphorylation to promote p53 binding to Sp1 (Fig. 7B). Ou *et al.* have reported that p53 C-terminal phosphorylation can also affect p53 C-terminal acetylation [64]. Thus, the phosphorylation status of p53 at residue 378 could also promote the modification of several residues within the p53 C-terminus to alter the binding between p53 and Sp1. In conclusion, TPA-induced phosphorylation of p53 residue 378 increases the repression of K14 expression by enhancing the binding between p53 and Sp1.

## Materials and Methods

### Cell Culture

C9 were obtained as described in references [15], [16], and H1299 cells were obtained from the American Type Culture Collection (ATCC), Cat. No. CRL-5803. C9 cells and H1299 cells were maintained in RPMI 1640 medium supplemented with 10% fetal bovine serum and 1% penicillin G–streptomycin sulfate. PHFK cells were maintained in MEPICF medium (Invitrogen M-EPICF-500) supplemented with 1% human keratinocyte growth supplement (HKGS) (Invitrogen S-002-5) and 1% penicillin G–streptomycin sulfate. All cells were incubated at 37°C in a humidified 5% CO_2_ incubator.

### p53 Mutant Expression Vector Constructs

The ΔTAp53, p53Δ364-393, p53Δ293-393, p53 248W, p53 378A, p53 378D, and ΔTAp53-Flag clones were created using the Phusion Site-Directed Mutagenesis kit according to the manufacturer’s instructions (Finnzymes F-541). The following primers were used for the site-directed mutagenesis reactions: ΔTAp53 CTCCATGGCAGTGACCCGGA and CCTACACCGGCGGCCCC; p53Δ364-393 TGAGGATCCACTAGTAACGGCCGC and CCTGCTCCCCCCTGGCTC; p53Δ293-393 TGAGGATCCACTAGTAACGGCCGC and TTTCTTGCGGAGATTCTCTTCCTCTGTG; p53 248W CATGAACCGTGGGCCCATCCT and CCGCCCATGCAGGAACTGTTAC; p53 378A GCTCGCCATAAAAAACTCATGTTCAAGACAGA and GGTAGACTGACCCTTTTTGGACTTCAGG; p53 378D GACCGCCATAAAAAACTCATGTTCAAGACAGA and GGTAGACTGACCCTTTTTGGACTTCAGG; ΔTAp53-Flag GATGACGACAAGTGAGGATCCACTAGTAACGGCCGCCA and GTCTTTGTAGTCGTCTGAGTCAGGCCCTTCTGTCTTGAA. All constructs were verified by direct DNA sequencing.

### Differentiation Drug Treatment

C9 cells were grown in six-well tissue culture dishes to 80% confluence and then were transfected with 2 µg of pCMV-cDNA 3.1, pcDNA 3.0 ΔTAp53, control siRNA (5'-UUCUCCGAACGUGUCACGUTT-3′) or p53 siRNA (5′-CUACUUCCUGAAAACAACGTT-3′). Twenty-four hours post-transfection, cells were treated with 100 ng/mL 12-O-tetradecanoylphorbol-13-acetate (TPA, Sigma P1585) or DMSO for 24 hours. Then, the cells were harvested in 0.2 mL of RIPA buffer containing phosphatase and protease inhibitor cocktails (Roche) or 1 mL of Trizol.

### p53 Inhibitor Treatment

PHFK cells were grown in six-well tissue culture dishes to 60% confluence and then were treated with 30 µM pifithrin-α (PFT-α, Sigma P4359) or DMSO for 24 hours. Following PFT-α treatment, cells were treated with 100 ng/mL TPA or DMSO for 24 hours. Then, cells were harvested in 0.2 mL of RIPA buffer containing protease and phosphatase inhibitor cocktails or 1 mL Trizol.

### Construction of Reporter Vectors with K14 Promoter Mutants

The full length K14 promoter (K14-p2000) was amplified from the pGem3ZK14 cassette and was subcloned into the pGL3 basic firefly luciferase vector (Promega). The K14-p269, K14-p187, K14-p160, K14-p160-SP1 mutant, and K14-p160-TATA mutants were created using the Phusion Site-Directed Mutagenesis kit according to the manufacturer’s instructions (Finnzymes). All constructs were verified by direct DNA sequencing. The following primers were used for the site-directed mutagenesis reactions: K14-p269 AGGGCTGGGACTCCCAGGGT and GGGCTAGCACGCGTAAGAGCTCG; K14-p187 AGAAAGCCCAAAACACTCCAAACAATGAG and GGGCTAGCACGCGTAAGAGCTCG; K14-p160; AGTTTCCAGTAAAATATGACAGACATGATG and GGGCTAGCACGCGTAAGAGCTCG; K14-p160-SP1 GAAAGTGCCAGAGGTACCCCCTACCCAT and CATTCCCCTTGGCTTTCATCACCC; K14-p160-TATA CCTACCCATGAGCCCAAAGCACTCGCATC and GGGCGGGTCTGGCACTTTCC.

### Luciferase Activity Assay

H1299 cells were grown in 12-well tissue culture dishes to 60% confluence and then were co-transfected with 0.5 µg of pcDNA 3.0 (Invitrogen), pcDNA 3.0 p53, pcDNA 3.0 ΔTAp53, pcDNA 3.0 p53Δ293-393, or pcDNA 3.0 p53 248W and 0.5 µg of pGL3 basic firefly luciferase (Promega) under the control of the wild-type K14 promoter or various mutant K14 promoters and 10 ng of pCMV renilla luciferase plasmid (Promega). Twenty-four hours post-transfection, cells were harvested in 0.25 mL of reporter lysis buffer and were subjected to a dual luciferase assay according to the manufacturer’s instructions (Dual-Luciferase Reporter Assay System, Promega). Firefly luciferase activity was normalized to renilla luciferase activity, and the data were presented as the mean ± standard deviation of three independent experiments, each performed in triplicate.

### Reverse Transcription–PCR (RT–PCR)

Total RNA was extracted using the Trizol reagent, and 1µg of RNA was reverse transcribed into cDNA using the SuperScript III Reverse Transcriptase cDNA Synthesis kit according to the manufacturer’s instructions (Invitrogen). Specific primer-paired amplification of ΔNp63α, K14, p21, and β-actin was measured. PCR reactions were performed using recombinant Taq polymerase under the following cycling conditions: 30 cycles (K14, p21, β-actin) or 35 cycles (ΔNp63α) at 95°C for 50 s, annealing (62°C for K14 and 60°C for ΔNp63α, p21, and β-actin) for 45 s, and 72°C for 2 min. Amplified products were analyzed on 1% agarose gels. The following specific primers were used: ΔNp63α (+) 5′–CCAGACTCAATTTAGTGAGC–3′ and (-) 5′–ACTTGCCAGATCATCCATGG–3′; K14 (+) 5′–GACCATTGAGGACCTGAGG A–3′ and (-) 5′–GGCTCTCAATCTGCATCTCC–3′; p21 (+) 5′–CTTTGTCACCGAGACACCAC–3′ and (-) 5′ GGCGTTTGGAGTGGTAGAAA–3′; β-actin (+) 5′–ACCATGGATGATGATATCGC–3′ and (-) 5′–TTGCTGATCCACATCTGCTG–3. The product sizes were as follows: ΔNp63α, 1453 bp; K14, 224 bp; p21, 311 bp; β-actin, 1079 bp.

### Real-time-RT-PCR

cDNA samples were pre-mixed with 2X Maxima™ SYBR Green qPCR Master Mix (Fermentas) and the following specific primer pairs: p53 (+) 5′–AGCGATGGTCTGGCCCCTCC–3′ and (-) 5′–GCGGCTCATAGGGCACCACC–3′; p63 (+) 5′–GGAAAACAATGCCCAGACTC–3′ and (-) 5′–CGCGTGGTCTGTGTTATAGG–3′; K14 (+) 5′–CCTCTCCTCCTCCCAGTTCT–3′ and (-) 5′–GGACACCACCTTGCCATC–3′; p21 (+) 5′–GGAAGACCATGTGGACCTGT–3′ and (-) 5′–GGCGTTTGGAGTGGTAGAAA–3′; β-actin (+) 5′–GGCATGGGTCAGAAGGATT–3′ and (-) 5′–GAAGGTGTGGTGCCAGATTT–3′. Real-time PCR product sizes were as follows: p53, 115 bp; ΔNp63α, 124 bp; K14, 112 bp; p21, 146 bp; β-actin, 135 bp. The real-time PCR analysis was conducted using the Applied Biosystems 7500 machine, and the following cycling parameters were used: 50°C for 2 min, 95°C for 10 min, followed by 40 cycles that consisted of denaturation at 95°C for 15 s and annealing/extension at 60°C for 60 s. Data acquisition and analysis were conducted using the ABI Prism 7500 SDS software.

### Western Blot Analysis

Twenty micrograms of total cellular protein extract was separated on 10% SDS–polyacrylamide gels. Proteins were transferred onto a polyvinylidene difluoride (PVDF) membrane and then were blocked with 3% skim milk in phosphate-buffered saline and Tween-20. Membranes were probed using specific antibodies for N-terminal p53 (Pab1801 mouse monoclonal, Santa Cruz sc-98), C-terminal p53 (Pab122 mouse monoclonal, Lab Vision), p63 (A4A mouse monoclonal, Sigma), K14 (C-14 goat polyclonal, Santa Cruz sc-17104), p21 (12D1 rabbit polyclonal, Cell Signaling), β-actin (rabbit polyclonal, Lab Vision), or Sp1 (H-225 rabbit polyclonal, Santa Cruz sc-14027). Goat anti-mouse, goat anti-rabbit, or donkey anti-goat immunoglobulin G (IgG) conjugated with horseradish peroxidase was used as the secondary antibody (Santa Cruz). Chemiluminescent signals were detected using an enhanced chemiluminescence (ECL) kit (Amersham).

### Co-immunoprecipitation

Cells were scraped into RIPA buffer containing protease and phosphatase inhibitor cocktails. After removing cellular debris by centrifugation, the pre-cleared extract was incubated with Sp1 (H225), p53 (PAb1801) or phospho-Ser378 p53 antibody and 20 µL of protein A/G agarose (Santa Cruz) for 2–3 h at 4°C. Beads were collected by centrifugation and were washed three times in 10S buffer (50 mM HEPES, pH 7.5, 150 mM NaCl, 0.2% NP-40, 0.1% Triton X-100, and 0.01% SDS). Bound proteins were eluted in 20 µL of SDS–PAGE sample buffer and were detected by using N-terminal p53 (Pab1801) or Sp1 (H225) antibody by western blot.

### DNA Affinity Precipitation Assay

Two hundred micrograms of nuclear extract was prepared from 1×10^7^ H1299 cells transfected with 32 µg of p53Δ364-393 or p53Δ293-393 plasmid. The nuclear extracts were pre-cleared by incubation with 40 µL of streptavidin–agarose beads (4%) in a 50% slurry at 4°C for 1 h with rotation. Then, the nuclear extracts were centrifuged at 4000 g for 1 min at 4°C, and the supernatant was collected as the pre-cleared nuclear extract. The following annealing probes were used: wild-type SP1 (+) Biotin-5′-CCAGACCCGCCCCCTA-3′ and (-) 5′-TAGGGGGCGGGTCTGG-3′; and mutant SP1 (+) Biotin-5′-CCAGAGGTACCCCCTA-3′ and (-) 5′-TAGGGGGTACCTCTGG-3′. The binding reaction was performed by mixing the pre-cleared nuclear extract with the annealing probe (0.3 nmol), 5X binding buffer (100 mM HEPES; pH  = 7.6, 5 mM EDTA, 50 mM (NH_4_)_2_SO_4_, 5 mM DTT, 1% Tween 20 (w/v), and 150 mM KCl), 10 µL of sonicated salmon sperm DNA (1 µg/µL), and 40 µL of streptavidin–agarose beads (4%) in a 50% slurry to a final volume of 1 mL. The mixture was incubated at room temperature for 1 h with rotation. The beads were washed three times with 1X PBS and 0.1% NP-40. Bound proteins were eluted with 20 µL of 5X loading dye and then were separated by sodium dodecylsulfate polyacrylamide gel electrophoresis (SDS–PAGE) followed by western blot analysis. The bound proteins were probed using anti-p53 antibody (p53 Ab-2, Lab Vision). Ten micrograms of nuclear protein was mixed with 20 µL of 5X loading dye and was included as the 5% input sample.

### Chromatin Immunoprecipitation (ChIP)

C9 cells were grown to 90% confluence in a 10-cm culture dish, and then chromatin immunoprecipitation was performed according to the manufacturer's instructions (EZ ChIP kit, Upstate) using anti-IgG (rabbit, Sigma), anti-Sp1 (sc-14027 rabbit polyclonal, Santa Cruz) or anti-p53 (sc-98 mouse monoclonal, Santa Cruz) antibody. DNA eluted from precipitated complexes was amplified for various fragments of the K14 promoter by PCR using the following primers: K14 promoter (-689 to -551): forward (5′-GATGTGAGATCCTCACCATAGG-3′) and reverse (5′-CTGTGCTGAGAAGTCTGTCC-3′); and K14 promoter (-220 to -5) forward (5′-CACCTCCCCCTGTGAATCAC-3′) and reverse (5′-GCAAAGGGATGCGAGTGCTT-3′). PCR reactions were performed using the following cycling conditions: 35 cycles at 95°C for 40 s, annealing at 60°C for 40 s, and extension at 72°C for 40 s. PCR products were loaded onto 2% agarose gels.
